# RFX6 expression is central to the development and function of the neuroendocrine compartments of the pancreas and intestine and strongly affects diabetes risk

**DOI:** 10.1007/s13340-025-00867-1

**Published:** 2026-02-17

**Authors:** Thomas S. R. Bate, Yuanhao Huang, Xin Luo, Diane C. Saunders, John T. Walker, Vivek Rai, Stephen C. J. Parker, Jie Liu, Marcela Brissova

**Affiliations:** 1 Division of Diabetes, Endocrinology, and Metabolism, Department of Medicine, Vanderbilt University Medical Center, Nashville, TN 37232 USA; 2 Gilbert S. Omenn Department of Computational Medicine & Bioinformatics, University of Michigan, Ann Arbor, MI 48109-2218 USA; 3 Department of Pediatrics, Feinberg School of Medicine, Northwestern University, Chicago, IL 60611 USA; 4 Division of Endocrinology and Diabetes, Department of Pediatrics, Children’s Hospital of Philadelphia, Philadelphia, PA 19104 USA; 5 Department of Computer Science & Engineering, University of Michigan, Ann Arbor, MI 48109-2121 USA

**Keywords:** RFX6, Pancreas, Monogenic diabetes, Maturity onset diabetes of the young, Type 2 diabetes, Islets of langerhans

## Abstract

Transcription factors are central to the developmental and functional regulation of cells through co-ordination of gene expression via interaction with the genome. RFX6 is a winged-helix transcription factor whose expression is highly specific to the pancreas and gastrointestinal tract. Recent developments have highlighted an association between RFX6 and Type 2 Diabetes (T2D), which affects over 500 million people throughout the world. RFX6 controls development in both the pancreas and the gastrointestinal tract, where it is required for the differentiation of islet endocrine and enteroendocrine cells. Coding and non-coding *RFX6* variants have been associated with syndromic neonatal diabetes (Mitchell-Riley Syndrome), Maturity Onset Diabetes of the Young (MODY), and T2D. Given the central position of RFX6 in pancreatic development and diabetes, understanding in more detail the regulatory role of RFX6 in different cell types and at different stages of development may open avenues towards patient-specific diabetes treatment and prevention. In this article, we review the literature surrounding RFX6 with respect to its role in development and diabetes pathogenesis.

## Introduction

Transcription factors that regulate the development and function of the endocrine pancreas are critical determinants of diabetes pathophysiology, a disease of multiple forms but broadly characterized by a loss of functional β cell mass, resulting in systemic glucose dysregulation. Diabetes affects 500 million lives and constitutes a significant burden on healthcare systems worldwide [1]. Heterogeneity is observed not only between the major classifications (e.g., Type 1 Diabetes (T1D), Type 2 Diabetes (T2D)) but also within these subtypes, where differences in environments and genetics can make it difficult to individualize treatment. Understanding how individual molecular pathways may contribute to specific pathogenic processes is crucial for delivering personalized treatments that are increasingly acknowledged as the future of precision medicine for diabetes.

Recent findings surrounding the winged-helix transcription factor RFX6 have revealed strong associations with multiple forms of diabetes pathogenesis. RFX6 was discovered in the human genome in 2008 and, unlike other members of the RFX family (RFX1-8), its expression is restricted mainly to the pancreas and gastrointestinal tract [2, 3]. Soon after the discovery of RFX6, it emerged as a major regulatory factor involved in pancreatic islet development, and *RFX6* loss-of-function variants in coding DNA regions were found to cause Mitchell-Riley syndrome, an autosomal recessive condition resulting in intestinal atresia, pancreatic hypoplasia and neonatal diabetes [4]. More recently, studies have revealed an association between RFX6 and T2D, where a genetic network under the regulation of RFX6 strongly predisposes T2D risk [5–7]. In this review, we will discuss the current scientific literature with respect to RFX6 in the context of pancreas and gastrointestinal development, function in mature endocrine cells, and diabetes.

Our approach to this review employed a novel method of literature search using the Genomic Literature Knowledge Base (GLKB), a graph-based database that has guided the selection of literature for review. We hope that this example is informative and encourages readers to explore the use of such platforms for their own research purposes.

## Identification and analysis of RFX6-related biomedical concepts

We employed GLKB to systematically identify and analyse biomedical concepts associated with RFX6 [8]. The analysis was based on concept co-occurrence in PubMed articles, utilizing Jaccard similarity coefficients. Initially, we identified the 20 most closely related entities in each of four categories: genes, chemicals, diseases, and SNPs, yielding 88 distinct biomedical concepts. This initial concept set was expanded by identifying the top 100 biomedically relevant concepts that showed significant similarity to the original 88 concepts.

**Fig. 1:**
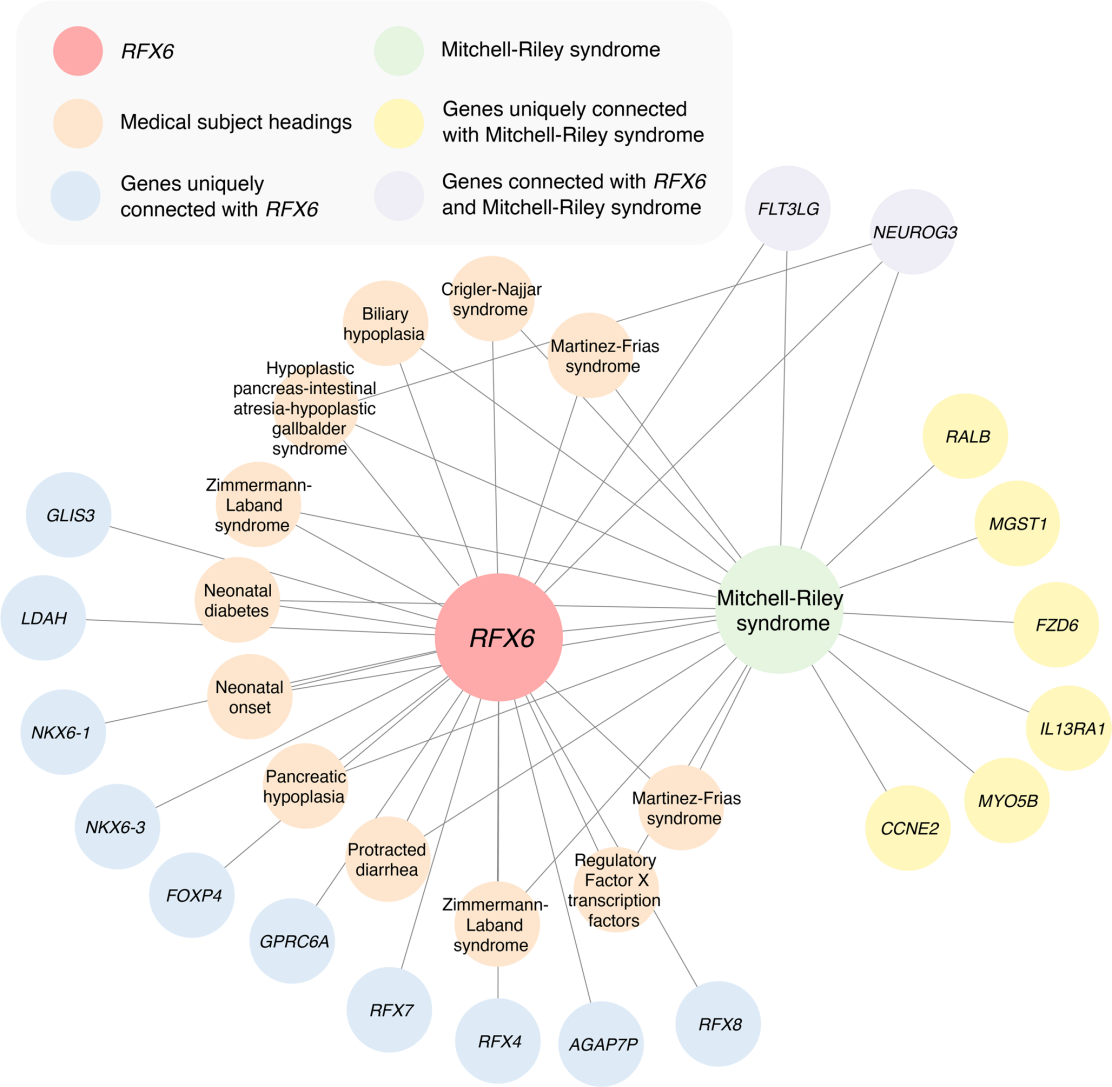
Utility of the Genomic Literature Knowledge Base (GLKB) to identify literature relevant to *RFX6*. This graph representation of *RFX6* related ontological terms shows relevant terms (e.g. Mitchell-Riley Syndrome) connected to *RFX6* within GLKB. Nodes represent ontological terms and edges represent connections between terms found within abstracts on PubMed. With this visualization one can see both direct and indirect associations between ontological terms and *RFX6*. Selection of relevant nodes for this review returned PubMed abstracts that were subsequently sorted based on topic and included in the review

Network analysis of these 188 biomedical concepts revealed distinct groups with biological relevance. From these, 14 groups were manually selected for detailed investigation, from which 63 relevant articles were retrieved using GLKB, shown in Fig. 1. These articles specifically documented the interactions between RFX6 and other concepts within the groups. The relationships described in these articles were subsequently analyzed and summarized using GPT4.

The analysis revealed varying degrees of biological relevance among the identified groups. Some groups successfully captured well-established scientific associations, such as the relationship between RFX6 and Maturity-Onset Diabetes of the Young (MODY), and RFX6 and Type 2 Diabetes (T2D). Other groups demonstrated other biologically relevant associations, such as the connection between RFX6 and prostate cancer (including concepts like C2orf43, RFX6, prostate cancer, and rs10090154), which were not selected for this review. Notably, the groups with more substantial relevance to our topic were typically supported by a larger body of literature (63 articles), while those with weaker biological associations were supported by fewer publications (5 articles). These findings underscore the continued importance of human expertise in validating and interpreting computationally derived concept associations.

## Organogenesis of the pancreas and intestine

### Developmental expression patterns of RFX6 in model organisms

Two seminal studies published in early 2010, by *Smith *et al*.* and *Soyer *et al*.*, screened NGN3 + pancreatic endocrine progenitors in search of transcription factors regulating islet formation in mice [4, 9]. These screens independently identified RFX6 as a novel transcription factor expressed in early endocrine cell development. Subsequent *in-situ* hybridization and immunohistochemical analyses in mouse embryos demonstrated the appearance of RFX6 early in the definitive endoderm and continued expression within gut endoderm, where newly forming pancreatic progenitors showed partially overlapping expression of RFX6 and PDX1. Upon formation of the nascent pancreatic bud RFX6 + cells were scattered, where RFX6 + cells were separate from PDX1 + and PTF1A + pancreatic progenitor cells. RFX6 expression was eventually restricted to the NGN3 + and post-NGN3 + endocrine progenitors around the peak of endocrine differentiation at E15.5. *Smith *et al*.* demonstrated the absence of RFX6 expression in the pancreata of *Ngn3*^−/−^ mouse embryos at E13.5, and *Soyer *et al*.* showed that RFX6 expression was unaffected by the knockout of developing islet markers ARX, PAX4, and NEUROD1. These analyses placed RFX6 expression in mice downstream of NGN3 + expression during islet development and upstream of or in parallel with other key developmental transcription factors. Antisense morpholino-mediated knockdown of RFX6 expression in Zebrafish embryos by *Soyer *et al*.* showed that RFX6 was required for the production of fully differentiated islet endocrine cells. Similarly, *Smith *et al*.* showed that mice homozygous for a loss-of-function *Rfx6* mutant showed a complete lack of INS + , GCG + , and SST + cells; however, cells positive for the neuroendocrine marker Chromogranin A remained. This finding led to the discovery of *RFX6* homozygous variants as the causal factor in Mitchell-Riley syndrome, discussed later in this review.

In a later study on developing Xenopus embryos, *Pearl *et al*.* showed similar patterns of RFX6 expression with broad expression in the early anterior endoderm and eventual restriction within the developing pancreas, reflecting Xenopus expression patterns for other endocrine progenitor markers [10]. Targeted antisense morpholino-mediated knockdown of RFX6 in the anterior endoderm of Xenopus led to a reduction in the expression of the developing early foregut and pancreas markers HNF6, FOXA2, PTF1A, and PDX1 (developmental stages NF25-35). By developmental stage NF42, expression of PDX1 and PTF1A in the developing dorsal and anterior pancreas was almost undetectable, and endocrine hormone markers insulin, glucagon, and somatostatin were greatly reduced. Expanding the investigation by microarray mRNA analysis of RFX6 loss-of-function Xenopus embryos, *Pearl *et al*.* showed multiple pancreas associated genes to be down-regulated at varying points between developmental stages NF30—NF44.

More recently, efforts made to characterize the RFX6 regulatory network have highlighted the role of RFX6 in endocrine cell development. *Cheng *et al*.* conducted chromatin immunoprecipitation targeting *Rfx6* in adult mouse pancreata and sequenced the bound genomic DNA fragments (ChIP-Seq) to identify targets of RFX6 [11]. This confirmed *Pdx1* and *Neurod1* as direct binding targets of RFX6 and revealed novel target genes previously associated with islet endocrine cell development, such as *Hnf1a* and *Nkx6-1*, supporting the notion that RFX6 regulates a gene network required for proper islet endocrine cell development.

### Manipulation of RFX6 expression in stem cell differentiation models of pancreatic development

Aside from developmental studies in whole organisms, RFX6 expression has also been explored in stem cell models of pancreatic differentiation. Such models utilize multi/pluripotent stem cells derived from embryos, mesenchymal tissues, or adult somatic cells induced to exhibit pluripotency. Multistep differentiation protocols allow researchers to direct the differentiation of these stem cells towards intended cell lineages, discretely modelling the process of development in vitro. Genomic editing of the initial stem cell population thus allows for the interrogation of gene function at various stages through the modelled development process. Importantly, these stem cell models can be implemented using stem cells derived from human donors, offering the chance to probe developmental processes in human. One such study investigated the role of several known pancreatic cell lineage determinants, including RFX6, in four discrete stages of human embryonic stem cell (hESC) differentiation, up to a poly-hormonal β-like (PH-β) cell stage, using a doxycycline inducible CRISPR-Cas9 gene knockout system [12]. The authors found that *RFX6*^−/−^ mutant hESCs showed a large reduction of PDX1 + cells upon differentiation into an early pancreatic progenitor (PP) like stage, suggesting that RFX6 expression is required to define a significant pool of PDX1 + PP cells. In accordance with previous studies in model organisms, pancreatic endocrine hormone expressing cells in the *RFX6*^−/−^ mutant line were completely absent from the final PH-β cell differentiation stage. Another similar study, by *Aldous *et al*.,* also applied CRISPR-Cas9 to knockout *RFX6* in a model of islet development in iPSCs, modelling 6 stages of endocrine cell differentiation through to islet-like cells [13]. The authors observed that RFX6 expressing iPSCs showed co-expression of RFX6 and PDX1 during the posterior foregut (PF) stage, but RFX6 did not co-localize with PDX1+/NKX6.1+ cells at the PP stage. *RFX6*^−/−^ iPSCs were able to generate some PDX1 + /NKX6.1 + pancreatic progenitors, though there was a significant reduction, which was attributed to a reduction in PDX1 + cells during the prior PF stage. This suggests the possibility for a subset of PDX1 + /NKX6.1 + pancreatic progenitors that escape RFX6 regulation during development. The study showed reduced expression of islet development factors throughout the differentiation of *RFX6*^−/−^ iPSCs and proposed reduced catalase expression to cause increased apoptosis at the resulting differentiated islet-like organoid stage.

Given that *RFX6* homozygous and heterozygous variants had been associated with various forms of diabetes, some studies have explored pancreatic cell differentiation in stem cells derived from *RFX6* variant carriers. *Trott *et al*.* describe a consanguineous family where both parents carried a heterozygous *RFX6* nonsense variant (c.1129C > T). The authors collected skin fibroblasts from two of the deceased progenies, homozygous for the *RFX6* nonsense variant, and the healthy father who was heterozygous for the *RFX6* nonsense variant [14]. The skin fibroblasts were transformed into iPSCs and went through four differentiation steps from definitive endoderm to pancreatic progenitor stages. iPSCs carrying homozygous *RFX6* variants showed reduced PDX1 expression throughout the differentiation stages compared with the heterozygous *RFX6* mutant iPSCs. Comparison of both heterozygous and homozygous iPSCs to a wild-type H9 cell control showed a dose-dependent effect of *RFX6* on the differentiation of PDX1 + /NKX6.1 + pancreatic progenitors. Gene expression analysis indicated major changes in genes associated with pancreatic development, primarily beginning at the pancreatic endoderm, indicating that RFX6 is specifically required during the formation of the pancreatic endoderm. *Ibrahim *et al*.* developed two models of stem cell-differentiated islet organoids [15]. One utilized human embryonic stem cells (hESCs) from a donor with two functional *RFX6* alleles, and the other was an induced pluripotent stem cell model derived from fibroblasts obtained from a patient homozygous for the p.His293LeufsTer7 *RFX6* variant presenting with Mitchell-Riley syndrome. CRISPR gene editing was used in both models to generate *RFX6*^+*/*+^, *RFX6*^+*/*−^, and *RFX6*^*−/−*^ lines. In vitro endocrine differentiation of these lines confirmed the absence of RFX6 expression in *RFX6*^−/−^ lines and significantly reduced *RFX6* expression in the *RFX6*^+*/*−^ line, confirming haploinsufficiency due to the heterozygous variant. The emergence of PDX1 + /NKX6.1 + cells was significantly reduced in *RFX6*^*−/−*^ lines, however the *RFX6*^+*/*−^ and *RFX6*^+*/*+^ lines showed similar populations. By the islet-like stage, there was a complete absence of islet hormone-expressing cells, and the authors showed persistent upregulation of endocrine precursor markers SOX9 and NGN3, which was accompanied by increased apoptosis.

### Role of RFX6 expression in early endoderm patterning

Patients with Mitchell-Riley syndrome present with multiple gastrointestinal abnormalities in conjunction with a hypoplastic pancreas and neonatal diabetes, such as duodenal malrotation/atresia. To elucidate the role of RFX6 loss-of-function in the development of these abnormalities, iPSC lines have also been used as model systems. *Nakamura *et al*.* developed *RFX6*^*+/eGFP*^ heterozygous knock-in and *RFX6*^*eGFP/eGFP*^ homozygous knock-in/knock-out iPSC lines to study the effect on early endoderm development [16]. The parental *RFX6*^*+/+*^ hiPSC line switched on RFX6 expression upon differentiation into the primitive gut tube (PGT) stage in conjunction with master regulators, PDX1 and CDX2, of posterior foregut and mid-hindgut, respectively. This, however, lies in contrast to *Aldous *et al*.,* who did not detect RFX6 until the PF stage. RFX6 expression decreased almost linearly with the *RFX6*^*+/eGFP*^ and *RFX6*^*eGFP/eGFP*^ lines at the PGT stage, where RFX6 expression was absent in the *RFX6*^*eGFP/eGFP*^ line. PDX1 and CDX2 expression was roughly maintained at the PGT stage in the *RFX6*^*+/eGFP*^ line, however a marked reduction was observed in the *RFX6*^*eGFP/eGFP*^ line. Notably, expression of the anterior foregut marker, SOX2, was unaffected in *RFX6* knockout lines. These results suggest that RFX6 is a crucial regulator involved in endoderm patterning processes that lead to both pancreatic and intestinal development.

### RFX6 expression in the developing intestine

Furthermore, iPSCs derived from a patient with compound-heterozygous *RFX6* loss-of-function variants exhibited abnormal patterning when differentiated into intestinal organoids [17]. *Guillermo Sanchez *et al*.* showed a diverse set of abnormalities in the human *RFX6* loss-of-function intestinal organoids, where duodenal and intestinal endocrine cell development was impeded, which correlated with a loss of PDX1 expression. CRISPR-mediated correction of one of the faulty alleles restored the formation of the duodenal cell lineages, where PDX1 expression was increased. The presence of RFX6 during intestinal development had been shown previously, where RFX6 was implicated in the development of enteroendocrine cells. *Piccand *et al*.* revealed scattered RFX6 expression throughout the embryonic intestine, post gut endoderm, which co-localized with a subset of NGN3 + enteroendocrine progenitors [18]. *Ngn3-Cre* mediated knockout of RFX6 in mice resulted in decreased expression of multiple enteroendocrine hormones and ectopic expression of gastric-associated genes in the enteroendocrine cells. In a study designed for the identification of regulators of enteroendocrine development downstream of NGN3 expression in mice, transient RFX6 activity was observed in the differentiation of multiple enteroendocrine cell subtypes [19]. Subsequent RFX6 loss-of-function experiments showed that RFX6-deficient enteroendocrine progenitors failed to differentiate into L, K, and X enteroendocrine cell types.

### Key takeaways

Together, these findings demonstrate that RFX6 plays a role in various stages of pancreatic and intestinal development. The studies documented here have been conducted in different species and through multiple modalities, thus there are some conflicting reports regarding the order of events. The observations for pancreatic development in mice and humans to date are summarized in Table 1. RFX6 expression is observed as early as the primitive gut tube in human models and is gradually restricted to early pancreatic progenitors in the posterior foregut and also within the mid-hindgut. During the formation of the pancreatic bud RFX6 expression declines in the PDX1 + /NKX6.1 + pancreatic progenitors but continues in the developing NGN3 + endocrine progenitor population. Here, at least in mice, RFX6 expression is dependent on NGN3 expression suggesting that RFX6 expression undergoes regulatory changes during the early stages of the endocrine cell lineage development, possibly denoting a transition period in the upstream and downstream regulatory landscape of RFX6. RFX6 then proceeds to regulate the development of the entire endocrine lineage, where it is essential for the production of hormone-producing pancreatic islet cells.

**Table 1:** RFX6 is implicated in multiple stages of pancreatic development

Species	Development Stage					
	Primitive Gut Tube	Definitive Endoderm	Posterior Foregut	Pancreatic Progenitor	Endocrine Progenitor	Islets
Mouse (in vivo)		RFX6 appears	Continued RFX6 expression	RFX6 colocalizes with PDX1 + early pancreatic progenitors RFX6 separates from PDX1 + and PTF1A + pancreatic progenitors in developing pancreatic bud	RFX6 expression eventually restricted to NGN3 + endocrine progenitors *Ngn3*^−/−^ eliminates RFX6 expression in the developing pancreas at E13.5	RFX6 expressed in all endocrine lineages *Rfx6*^−/−^ results in absence of hormone expressing cells
Human (in vitro)	RFX6 appears (*Nakamura *et al*.*) *RFX6*^−/−^ reduces PDX1 and CDX2 expression		RFX6 appears (*Aldous *et al*.*) RFX6 colocalizes with PDX1 *RFX6*^−/−^ reduces PDX1 expression	RFX6 not expressed in PDX1 + /NKX6.1 + pancreatic progenitors *RFX6*^−/−^ and *RFX6*^+/−^ reduces PDX1 + /NKX6.1 + pancreatic progenitors, dose dependently		RFX6 expressed in all endocrine lineages *RFX6*^−/−^ results in absence of hormone expressing cells

This table summarizes the observations in human (in vitro) and mouse (in vivo) models with respect to RFX6 during pancreatic development stages: primitive gut tube, definitive endoderm, posterior foregut, pancreatic progenitor, endocrine progenitor, islets. RFX6 expression is observed as early as the primitive gut tube stage in human in vitro stem cell models, and is continually required for the formation of functional islet endocrine cells

## Function in adult islet endocrine cells and enteroendocrine cells

### Gene regulation and hormone secretion in islet endocrine cells

Given that RFX6 was highlighted as a major factor regulating endoderm patterning and pancreatic development, the question was raised regarding the role of RFX6 with respect to its persistent expression in islet cells and other cell types through to adulthood [20]. Two studies, published on the same day in the same journal, provided the first answers to this question in β cells [21, 22]. *Piccand *et al*.* developed an RFX6 knockout model in adult mouse β cells using an *Rfx6*^*fl/fl*^*;Ins1-CreER*^*T2*^ mouse. This allowed for adult β cell specific knockout of RFX6 which displayed positive immunolabeling for insulin protein, though reduced *Ins1* mRNA expression, upon RFX6 loss-of-function. Despite insulin protein expression in the adult RFX6 knockout mice, IPGTT and OGTT tests revealed significant glucose intolerance which could not be attributed to alterations in β cell mass. Plasma insulin responses to glucose injections and in vitro GSIS measurements in isolated islets showed significant reductions in insulin secretion under glucose stimulation. Gene expression analysis showed significantly reduced expression of known regulators of insulin secretion both upstream (GCK) and downstream (ABCC8, CACNA1A, CACNB2, CACNA1D, CACNA1C) of glucose metabolism. These findings were corroborated by CHIP-seq showing direct RFX6 binding at some of the aforementioned factors, and measurements of the ATP/ADP ratio and Ca^2+^ signaling, which both showed significant reductions in *Rfx6*^−/−^ β cells upon stimulation with glucose and KCl, respectively. Moreover, RNA-seq in *Rfx6*^−/−^ islets showed significant increases in β cell disallowed genes, pointing towards other potential mechanisms of β cell dysfunction upon RFX6 loss-of-function. *Chandra *et al*.* explored the role of RFX6 in adult human β cells via siRNA-mediated knockdown of RFX6 in the EndoC-βH2 cell line [22]. RFX6 knockdown resulted in significantly reduced *INS* mRNA levels, in agreement with the prior mouse model, and a corresponding reduction in insulin protein content. Reduced mRNA expression of major β cell ion channels was also observed, such as for *ABCC8*, *CACNA1A*, *CACNB2*, *CACNA1D*, and *CACNA1C*. It was shown through electrophysiological recordings that the reduced expression of L-type and P/Q-type Ca^2+^ channels was responsible for reduced transmembrane Ca^2+^ currents and decreased insulin granule exocytosis, where the reduction of L-type channels mediated the majority of the effect. The authors tested a known *RFX6* missense variant, which showed reduced activity at the insulin promoter compared with wild-type *RFX6*, explaining the reduction in insulin expression. Furthermore, the *RFX6* missense variant was unable to rescue L- and P/Q-type Ca^2+^ channel expression in *RFX6*^*−/−*^ EndoC-βH2 cells.

More recently, *RFX6* function in adult primary human islet cells has been interrogated using pseudoislet models to assess changes in islet cell function and gene expression. *Walker, Saunders, Rai *et al*.* tested shRNA-mediated global *RFX6* knockdown in a primary human pseudoislet model [7]. Dynamic insulin secretion measurements from *RFX6* knockdown pseudoislets showed significant reductions at basal (5.6 mM) and stimulating (16.7 mM) glucose concentrations and also under cAMP potentiation (via 3-isobutyl-1-methylxanthine, IBMX) and membrane depolarization (potassium chloride, KCl). Single-nucleus multiome profiling (snRNA-seq, snATAC-seq) revealed differentially expressed genes (DEGs) that were associated with ontological terms relevant to insulin secretion mechanisms, where significant snATAC-seq changes overlapped with RFX6 binding sites in the promoter regions of many of the DEGs. Notably, genes altered at the chromatin level with corresponding RFX6 motifs shared binding sites for the chromatin modifier activator protein (AP1). *RFX6* is expressed in all islet endocrine cells, where *Coykendall *et al*.* have observed high levels of *RFX6* expression in α cells compared with β cells [23]. The authors thus tested the effects of both global and α cell-specific knockdown of RFX6 on glucagon secretion and gene expression in a human pseudoislet model transplanted into the *NOD-scid-IL2Rγ*^*null*^ mouse. Upon induction of hypoglycemia via an intraperitoneal insulin tolerance test, it was shown that α cell-specific RFX6 knockdown pseudoislets secreted less glucagon than controls, however, with global RFX6 knockdown, glucagon secretion was unchanged. Electrophysiological recordings showed the same effect on glucagon secretion, whereby α cell-specific *RFX6* knockdown resulted in decreased glucagon exocytosis upon membrane depolarization and global knockdown did not. Analysis of transcriptomic changes, corroborated by *RFX6* CUT&RUN experiments, revealed some overlap between dysregulated RFX6 gene targets in α cells and β cells upon RFX6 loss-of-function, where VDCCs such as *CACNA1A* and *CACNA1C* were prominent. However, α cells and β cells exhibited distinct profiles of gene expression changes, suggesting differential roles of RFX6-regulated gene networks in α cells compared with β cells.

### Gut-derived incretin peptide secretion

As discussed earlier in this review, RFX6 plays a transient role in the differentiation of different enteroendocrine cell types, where RFX6 loss-of-function severely depletes mature enteroendocrine cell populations. RFX6 expression persists in some enteroendocrine cell types through to adulthood where it has been linked to the capacity of these cells to secrete peptide hormones, such as gastric inhibitory polypeptide (GIP) and glucagon-like peptide 1 (GLP-1). *Suzuki *et al*.* demonstrated direct binding of RFX6 to the GIP promoter region, and shRNA-mediated RFX6 knockdown in STC-1 enteroendocrine cell line resulted in decreased GIP expression and secretion [24]. Furthermore, the authors linked RFX6 to hypersecretion of GIP in mice fed with a high-fat diet (HFD), where RFX6 (and PDX1) expression increased significantly in the K-cells of HFD-fed mice. Conversely, *Richards *et al*.* showed that HFD fed mice exhibited significantly decreased expression of RFX6 in L-cells which was associated with impaired GLP-1 secretion [25]. *Rfx6* knockout in adult mouse enteroendocrine cells, by *Piccand *et al*.,* resulted in large reductions of enteroendocrine hormone expression (including *GIP* and preproglucagon mRNA), where mice deficient for RFX6 in enteroendocrine cells exhibited intestinal malabsorption, indicated by reduced feeding efficiency and increased food intake [18]. Intriguingly, quantitative trait GWAS analyses in a bovine cohort identified variants in a genomic window containing the *RFX6* locus that contributed to the additive genetic variance of food intake [26]. These data demonstrate an essential, and dynamic, role for *RFX6* in the regulation of mature enteroendocrine cells, which can be linked to phenotypic traits relevant to whole organism energy homeostasis.

### Key takeaways

There is a clear role for RFX6 in regulating mechanisms of hormone production and secretion in both islet endocrine cells and enteroendocrine cells. However, it is not clear whether the mechanisms that are regulated in each compartment are similar. In islets, both human and mouse models have shown that RFX6 regulates the expression of major islet hormones glucagon and insulin. The secretory mechanisms for these hormonse are regulated by RFX6 also, where genes implicated in glucose metabolism and membrane electrodynamics are affected by RFX6 loss-of-function. There are differences between α and β cells with regard to secretion mechanisms, and thus RFX6 loss-of-function shows distinct changes between the two cell types. In enteroendocrine cells, RFX6 is shown to regulate the expression of major incretin hormones GIP and GLP-1, where levels of RFX6 associate with diet. It is not clear from current observations whether the electrophysiology of these cells falls under the regulation of RFX6 as it does in islet endocrine cells. Nonetheless, the RFX6 regulatory network is tightly restricted to hormone-producing cells within the adult pancreas and gastrointestinal tract. These endocrine compartments communicate to coordinate blood glucose homeostasis, and thus it will be important to understand how diabetes associated *RFX6* variants may interact across these different hormone-producing domains.

## Diabetes

### Mitchell–Riley syndrome

In 2004, *Mitchell *et al*.* published a case study featuring five infants who presented with 'neonatal diabetes, hypoplastic or annular pancreas, jejunal atresia, duodenal atresia and gallbladder aplasia or hypoaplasia [27]. Immunohistochemical analysis of pancreas tissue from two of these cases showed cells labelled positive for the neuroendocrine marker chromogranin A. However, these chromogranin A + cells displayed a complete lack of insulin, glucagon and somatostatin-positive labelling, which provided an explanation for the rapid onset of diabetes after birth. Testing for genetic mutations in PLAGL1, GCK, and PDX1, known to cause other forms of neonatal diabetes, was negative in these patients, and it was postulated that a new autosomal recessive mutation may be the cause. The condition has since been referred to as Mitchell-Riley syndrome, and the diabetic phenotype is categorized as syndromic neonatal diabetes, due to the co-presentation of complex developmental abnormalities. A later investigation into transcription factors controlling islet development in mice downstream of the endocrine progenitor marker NGN3 identified RFX6-positive cells within the NGN3 + endocrine progenitor population and demonstrated eventual restriction of RFX6 to the islet endocrine hormone-positive cells by E18.5 [4]. The authors thus developed a homozygous *Rfx6* knockout mouse model to investigate its role in islet formation. They demonstrated near complete loss of islet hormone expression in the pancreas at E17.5 due to RFX6 loss-of-function. Despite absence of the islet endocrine hormones, the pancreata still contained clusters of chromogranin A + neuroendocrine cells. Given the striking similarity between the phenotype of homozygous *Rfx6* knockout mice and that of the patients described in *Mitchell *et al*.*, the authors tested the reported probands from the clinical study for homozygous mutations. Genetic sequencing confirmed homozygous missense, splicing, or frameshift *RFX6* mutations in the genomes of all of the selected probands. Since this discovery, several case studies have reported other novel homozygous *RFX6* mutations in cases presenting with syndromic neonatal diabetes [28–34]. Two notable case studies described biallelic *RFX6* mutations resulting in later childhood-onset diabetes and gastrointestinal abnormalities associated with Mitchell-Riley syndrome, such as duodenal atresia [33, 34]. One of these cases featured two compound heterozygous mutations and the other a homozygous missense mutation in *RFX6*. The relative delay of disease onset in these cases was suggested to be due to residual *RFX6* activity in at least one of the mutant alleles. Aside from the onset of diabetes, Mitchell-Riley syndrome results in multiple gastrointestinal abnormalities, as described in *Mitchell *et al. One case study describes protracted diarrhoea in two patients with Mitchell-Riley syndrome, which was associated with undetectable plasma GLP-1 and negative GLP-1 immunolabeling in the small intestine and colon [35]. The role of RFX6 in regulating enteroendocrine cell development and function has been described by several authors and provides a plausible explanation for loss of gut derived incretins due RFX6 loss-of-function in these patients. The convergence of findings from studies on Mitchell-Riley syndrome and pancreatic islet development laid the foundations for the understanding that functional RFX6 expression is required for proper formation of the gastrointestinal tract, pancreas and islets in both mice and humans, where complete RFX6 loss-of-function results in syndromic neonatal diabetes.

### Maturity onset diabetes of the young (MODY)

Targeted next-generation sequencing analysis by *Li *et al*.* on a Chinese cohort of 11 patients diagnosed with suspected MODY identified a heterozygous *RFX6* mutation (p.Ser217Phe) as a potential causal variant in one proband [36]. MODY typically arises through a monogenic autosomal dominant mutation and is commonly characterized by non-autoimmune-mediated diabetes onset post-infancy, though not exclusively. The diabetogenic potential of the heterozygous p.Ser217Phe *RFX6* variant was reasoned due to previous observations of a homozygous p.Ser217Pro *RFX6* mutation in a patient with syndromic neonatal diabetes. At a similar time, targeted screening by *Patel *et al*.* of a European cohort of 36 patients diagnosed with suspected MODY revealed two novel heterozygous nonsense *RFX6* variants in two of the probands [37]. Subsequent non-targeted whole-exome and whole-genome analysis in two replication MODY cohorts (n = 348, non-Finnish European; n = 80, Finnish European) established an association between three more novel heterozygous protein-truncating *RFX6* variants and MODY. Penetrance of diabetes in *RFX6* heterozygotes was shown to be less than that of known MODY-associated *HNF1A* and *HNF4A* variants, with diabetes onset in 27% of *RFX6* heterozygotes at 25 years. Separate analyses of individual case studies and large MODY cohorts in India, Turkey, Poland, Pakistan, USA and Japan have further confirmed a genetic association between heterozygous *RFX6* variants and cases of MODY [38–43]. Four studies explored familial inheritance of the heterozygous *RFX6* variants in a total of five probands, each showing a high degree of co-segregation of heterozygous *RFX6* variants with diabetes diagnoses, suggesting a causal association [37, 39, 42, 43]. Of note, the familial analyses identified two cases of gestational diabetes in carriers of heterozygous *RFX6* variants [37, 39]. Physiological and gastrointestinal aberrations associated with heterozygous *RFX6* variants have also been described. Non-diabetic *RFX6* heterozygotes in the Finnish cohort described by *Patel *et al*.* displayed lower glucose-stimulated insulin secretion upon an oral glucose tolerance test (OGTT) compared with population controls, and both fasting and OGTT-stimulated plasma GIP was significantly lower [37]. One case study also showed reduced post-prandial insulin, GIP, and GLP-1 in the serum of a MODY patient carrying a heterozygous *RFX6* variant [42]. Functional predictions about the nature of the described variants indicated that MODY-associated heterozygous *RFX6* variants frequently resulted in protein-truncation or frame-shift deletion, leading to *RFX6* haploinsufficiency. These findings together imply that the conserved function of a single *RFX6* allele may be sufficient for pancreas and islet development; however, *RFX6* haploinsufficiency results in a strong disposition for the development of diabetes later in life, where increased β cell demand (e.g., during pregnancy) and increasing age may factor into the onset of diabetes.

### Type 2 diabetes

The first data collection phase of the FinnGen research project analyzed whole genome sequencing and national health register data from 224,737 individuals to identify rare population-enriched variants associated with disease [5]. Fine mapping GWAS analysis in 15 common diseases, published by *Kurki *et al., identified a frameshift *RFX6* variant (p.His293LeufsTer7) strongly associated with T2D diagnoses. This variant was identified previously by *Patel *et al. in an independent Finnish cohort of MODY patients, where non-diabetic carriers showed reduced OGTT-stimulated insulin secretion along with reduced fasting and OGTT-stimulated GIP secretion [37]. Association of this *RFX6* variant with both MODY and T2D diagnoses highlights the clinical challenge with respect to the diagnosis of monogenic diabetes versus polygenic diabetes with high-risk variants, but nonetheless supports the diabetogenic potential of dysfunctional *RFX6* variants. A more recent analysis by *Ibrahim *et al. on a later data-freeze of the FinnGen study (n = 440,734 individuals) showed that carriers of the heterozygous p.His293LeufsTer7 *RFX6* variant were 80% more likely to develop T2D [15]. *Ibrahim *et al*.* went further to investigate the mechanism by which the *RFX6* p.His293LeufsTer7 variant predisposes T2D risk and tested the function of the p.His293LeufsTer7 *RFX6* variant in two stem cell models of RFX6^+/−^ and *RFX6*^*−/−*^ variant carriers. Staining of differentiated endocrine cells showed insulin + and glucagon + cells in the *RFX6*^+/−^ and *RFX6*^+*/*+^ lines but a complete absence of islet hormone staining in the *RFX6*^*−/−*^ lines. In vitro and in vivo functional analyses showed significant reductions in glucose-stimulated insulin secretion due to *RFX6*^+/−^ haploinsufficiency, despite having similar insulin content to *RFX6*^+/+^ lines. RNA-seq analyses also showed *RFX6*^+/−^ haploinsufficiency resulted in reduced expression of genes central to β cell function, including β cell maturation markers, potassium channels, and calcium channels. These studies of a heterozygous *RFX6* variant enriched within the Finnish population have demonstrated through both genetic and mechanistic investigations, that *RFX6* haploinsufficiency predisposes T2D risk likely because it is detrimental to the development and function of mature β cells.

As described above, a clear association between homozygous and heterozygous *RFX6* variants and multiple forms of diabetes has been established. These variants were identified from genetic associations with disease diagnoses. *RFX6* has independently been implicated in T2D pathophysiology via integrated multimodal approaches exploring molecular features in human islets aside from the genome. Analysis of chromatin structure by *Varshney *et al*.* in human islets showed, via ATAC-seq informed DNA footprinting, that RFX family TF binding sites were enriched for T2D associated SNPs identified previously through GWAS, where the SNPs were predicted to disrupt the motif specifically [6]. This provided the first evidence that genes falling under RFX family TF regulation may exhibit altered RFX TF binding and contribute to a disposition towards T2D. Single-cell multiome analyses by *Weng *et al*.*, integrating scRNA-seq and snATAC-seq data from isolated β cells from T2D patients, showed reduced expression of RFX6 to be correlated with an increase in a T2D pseudo-state model [44]. Furthermore, integrated molecular analysis by *Walker, Saunders, Rai *et al*.* sought to identify human islet-specific molecular events occurring in recent onset T2D through integration of hormone secretion, multiplexed imaging, transcriptomic, and genetic data from human control and early-T2D islet donors [7]. Transcriptomic analyses of isolated β cells from controls and early-T2D donors showed that RFX6 was significantly down-regulated in T2D β cells, reflecting the result from *Weng *et al*..* Construction of weighted gene co-expression networks (WGCNA) identified RFX6 as a hub gene at the center of a highly connected genetic network in β cells. This genetic network correlated with reduced first-phase glucose-stimulated insulin secretion, a hallmark of T2D islet dysfunction, and second-phase glucose-stimulated insulin secretion. Integration of T2D GWAS data showed that genes within this genetic network were highly enriched for T2D-associated SNPs. Mendelian randomization on known *RFX6* expression quantitative trait loci (eQTLs) in the UK Biobank cohort (n = 423,698 controls, n = 19,119 T2D) confirmed loci associated with decreased RFX6 expression in islets to be causally associated with T2D risk. This implicated non-coding *RFX6* variants, which affect RFX6 expression in the development of T2D, highlighting an additional component of *RFX6*-mediated T2D genetic risk. This multimodal approach connected known T2D GWAS variants through an *RFX6*-regulated gene network and identified novel non-coding *RFX6* variants causally associated with diabetes development, placing RFX6 at the center of a complex genetic network that increases the risk of T2D.

### Key takeaways

Since the discovery of *RFX6* loss-of-function variants as the causal factors in Mitchell-Riley syndrome, *RFX6* variants have been implicated in multiple forms of diabetes. The classification of diabetes cases that associate with *RFX6* variants is highly dependent on the functional nature of the variants, Homozygous *RFX6* loss-of-function variants cause syndromic neonatal diabetes due to impaired early development of the pancreas. Heterozygous *RFX6* loss-of-function variants allow for supposedly normal pancreatic development; however, there is a strong disposition for the development of diabetes later in life. T2D diagnoses are also associated with heterozygous *RFX6* loss-of-function variants, however non-coding *RFX6* variants and other gene variants that are embedded in the *RFX6* regulatory landscape have also been implicated in T2D development. It is clear that mechanisms of diabetes pathogenesis are closely related to *RFX6* activity, and understanding these mechanisms in more detail may reveal targets for diabetes treatment and prevention.

## Summary and future outlook

Our literature review has utilized a novel search platform, GLKB, to source literature that was relevant to our intended review goals. GLKB extracts key word terms from abstracts that reflect those found in various biological ontology databases and is then capable of connecting abstracts within a graph structured database. Organizing the literature in such a manner means that a connectivity graph could be visualized, connecting RFX6 to other ontological terms. Upon visualization of the RFX6 connectivity graph, it was clear that certain ontological terms connected with RFX6 would group together in separate sub-networks or ‘modules’. Our goal was to review the literature surrounding RFX6 with respect to its role in the pancreas and diabetes. With this we were able to select relevant modules from the connectivity graph and retrieve the associated literature where RFX6 was mentioned. This approach greatly accelerated the initial search process and gave a visual overview of the current knowledge landscape. Not only did this accelerate the knowledge aggregation process, but also drew attention to literature that could easily have been overlooked. For example, two studies reviewed here describe a connection between RFX6 and feeding efficiency. One of these studies focuses on a bovine cohort specifically studied in the context of feeding efficiency. Though we can only comment after the fact, it seems likely that such a study would be overlooked when conducting a literature search using traditional search engine technologies. The ever-growing amount and complexity of biological research articles makes comprehension of the underlying patterns increasingly burdensome. As we have demonstrated here, tools such as GLKB can assist with this process and, with the increasing abilities of artificial-intelligence, should eventually allow researchers to accelerate the generation of new hypotheses to test.

**Fig. 2:**
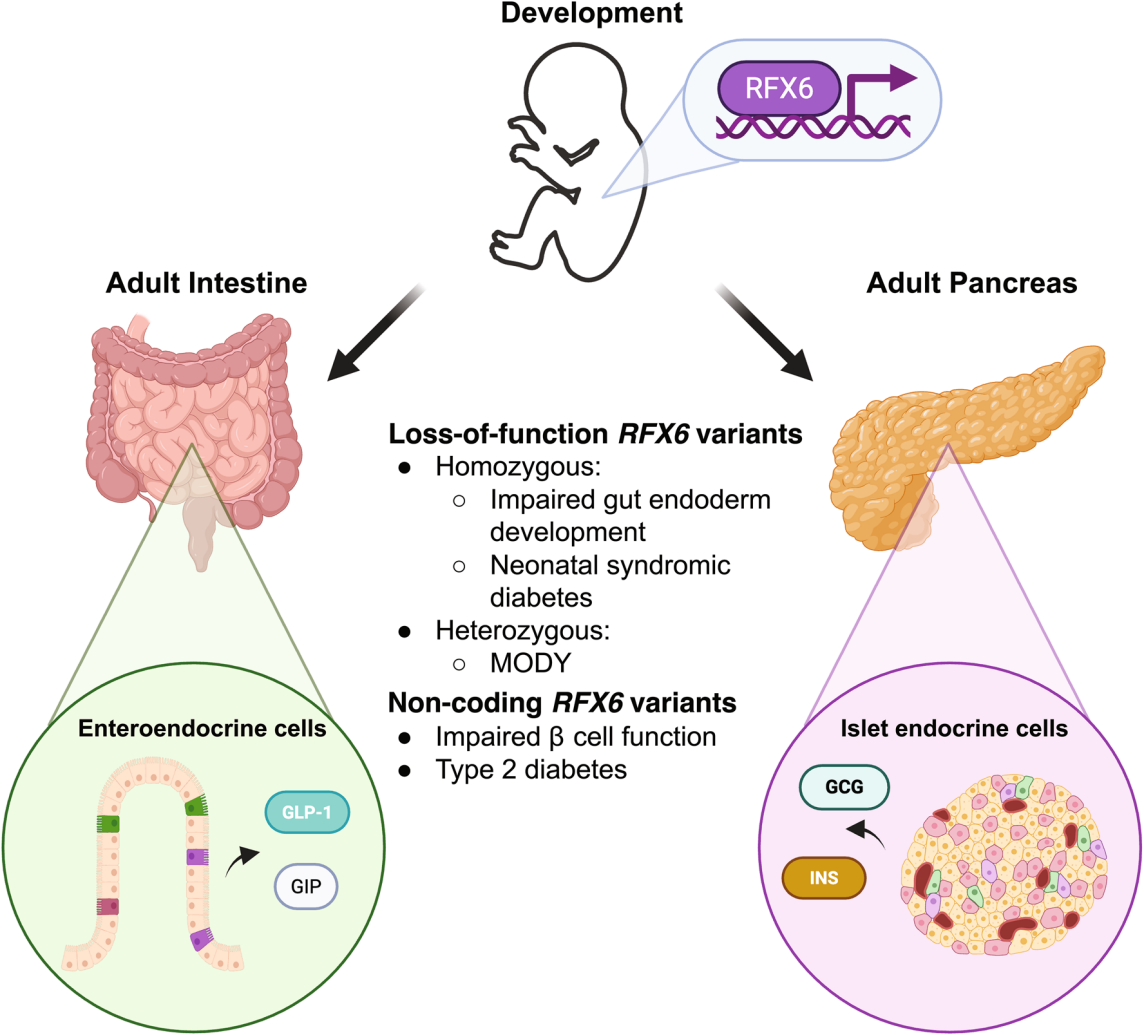
RFX6 regulates development of islet endocrine and enteroendocrine cells and associates with diabetes through coding and non-coding variants. *RFX6* instructs the development of the pancreas and gastrointestinal tracts from early stages through to islet endocrine and enteroendocrine cell differentiations. Coding and non-coding *RFX6* variants associate with diabetes of various forms

It is clear from the literature that RFX6 is a central component of the development and function of the gastrointestinal system, this is summarized in Fig. 2. RFX6 controls genetic networks in different phases of development, from patterning processes in the early gut endoderm to differentiation of islet endocrine cells and intestinal enteroendocrine cells, where its expression persists to maintain function. This underscores its capacity for inducing Mitchell-Riley syndrome upon complete loss-of-function, where multiple gastrointestinal abnormalities are presented in conjunction with neonatal diabetes. The multiphasic expression pattern of RFX6 indicates context-specific regulatory roles for RFX6, similar to other developmental islet transcription factors that persist in mature islets. During pancreatic islet development, RFX6 expression appears to be downstream of NGN3 (in mice) within the endocrine progenitor cells, yet NGN3 does not appear to regulate RFX6 during the earlier endoderm patterning processes, where RFX6 appears to regulate the development of a portion of PDX1 pancreatic progenitors. PDX1 is a significant factor that controls the development of the whole pancreas, where PDX1 loss-of-function can result in pancreatic agenesis [45]. Thus, it is important to understand the processes that lead to PDX1 expression prior to pancreatic bud formation, and it is apparent that RFX6 may play a significant and under-explored role. Further investigation into the specific function of RFX6 in endoderm patterning processes and the processes leading to the initiation of RFX6 expression will be an important step in defining early events during pancreatic development. Moreover, given that RFX6 expression falls downstream of NGN3 expression during development of the endocrine lineage in mice, this suggests a potential period of transition between the pancreatic progenitor and endocrine progenitor stages. Though not yet confirmed in human models, here the regulatory role of RFX6 may undergo significant change. Understanding in more detail the function of RFX6 at different developmental stages may provide further insight into the diabetogenic mechanisms behind diabetes associated *RFX6* variants.

Apart from developmental processes, RFX6 plays an essential role in the active regulation of function in mature islet endocrine and enteroendocrine cell types. In human α cells and β cells reduced RFX6 activity results in decreased expression and secretion of glucagon and insulin, respectively. Similarly, in enteroendocrine cells, RFX6 has been implicated in dynamic changes of hormone expression and secretion, such as GIP and GLP-1. Interestingly, RFX6 expression seems to be responsive to environmental exposures such as the HFD in enteroendocrine cells, it is not clear whether such responsiveness is observed within islet endocrine cells and should warrant further investigation. In islet cells, RFX6 directly regulates pathways that are crucial to hormone exocytosis. Common to both α and β cells is the direct regulation of L and P/Q-type VDCCs, which are responsible for the major influx of Ca^2+^ into the cytosol that triggers exocytosis of hormone-containing granules upon membrane depolarization. α and β cells lacking RFX6 activity are unable to generate sufficient cross membrane Ca^2+^ currents and so exocytosis of hormone granules is impeded. Though there is a link between RFX6 expression and hormone secretion in enteroendocrine cells, it is not clear whether a similar mechanism lies behind this association. More remains to be discovered regarding the mechanisms that are regulated by RFX6 in mature islet endocrine cells and enteroendocrine cells; however, it seems that there is a conserved role surrounding the production and secretion of the peptide hormones in different gastrointestinal neuroendocrine compartments that regulate whole-organism energy homeostasis.

Variants in *RFX6* have been associated with three major forms of diabetes, including neonatal diabetes, MODY and T2D. The severity and onset of diabetes is dependent on the extent to which RFX6 function is altered. Complete loss-of-function causes neonatal diabetes but heterozygous loss-of-function variants and non-coding variants in *RFX6* regulatory regions lead to less severe forms of MODY and T2D. Additional complexity arises when considering targets of RFX6 and their corresponding diabetes-associated variants, where *RFX6* targets are enriched for T2D-specific variants in β cells. However, one common observation is that the magnitude of *RFX6* expression is reduced in all forms of associated diabetes. On the condition that there is at least one functional *RFX6* allele, it is apparent that the pancreas can develop with a sufficient pool of islet endocrine cells. Precisely where and when the reduced magnitude of *RFX6* expression introduces vulnerabilities related to the pathogenesis of diabetes will need to be well defined in order to assist in identifying mechanisms that can be targeted for the prevention or treatment of diabetes. There is considerable evidence in this regard with respect to β cells but, as laid out in this review, there are other cell types relevant to diabetes that rely on *RFX6* to maintain function. For example, there is evidence that secretion of GIP and GLP-1 from enteroendocrine cells can be disrupted in *RFX6* MODY patients. This is reflected in findings from model organisms that show *RFX6* is related to the secretion of these incretin hormones. Given the rise of GLP-1 receptor agonists used to treat T2D and obesity, it remains plausible that disruptions in incretin hormone secretion may factor into the predisposition for diabetes development. Because non-coding *RFX6* variants that lie within the regulatory region of *RFX6* have been associated with T2D development, it could be speculated that dysregulation of RFX6 in the enteroendocrine system may impose compound effects on β cell function, on top of the β cell-intrinsic defects that have been demonstrated.

Overall, *RFX6* is a central regulatory factor in the pancreatic islets and perhaps the wider gastrointestinal system. *RFX6* activity is essential for the development of the hormone-producing pancreatic islets and intestinal enteroendocrine cells, and disruptions in *RFX6* activity can lead to several forms of diabetes. Further research should focus on how knowledge of variants that influence *RFX6* expression and its targets can be applied in a clinical setting and help with the potential prevention and treatment of diabetes.

## Data Availability

A data availability statement would not be applicable here as we have not generated any data. The article is a literature review and all relevant data and information can be obtained through the cited articles.
